# Diverticulosis and Diverticulitis on YouTube: Is Popular Information the Most Reliable?

**DOI:** 10.7759/cureus.64322

**Published:** 2024-07-11

**Authors:** Maverick H Johnson, Goutham A Nair, Courtney K Mack, Sean O'leary, Chris J Thang, Rui-Min D Mao, Nikhil R Shah, Uma R Phatak

**Affiliations:** 1 Surgery, University of Texas Medical Branch, Galveston, USA; 2 Psychiatry, University of Texas Medical Branch, Galveston, USA; 3 Internal Medicine, University of Texas Medical Branch, Galveston, USA; 4 Neurosurgery, University of Texas Medical Branch, Galveston, USA; 5 Dermatology, University of Texas Medical Branch, Galveston, USA; 6 General Surgery, University of Texas Medical Branch, Galveston, USA

**Keywords:** patient resources, social media, youtube, diverticulitis, diverticulosis

## Abstract

Background

Patients utilize online health information to inform their medical decision-making. YouTube is one of the most popular media platforms with abundant health-related resources, yet the quality of the disseminated information remains unclear. This study aims to evaluate the quality and reliability of content pertaining to diverticulosis and diverticulitis on YouTube.

Methods

One author queried the terms “diverticulosis,” “diverticulitis,” “acute diverticulitis,” and “chronic diverticulitis” on YouTube. The first 50 videos per search were selected for analysis. Duplicates, non-English videos, or procedural content were excluded. Video characteristics including view count, likes, comments, duration, days since upload, view ratio, video power index, and video sources (professional organizations (POs), health information websites (HIWs), and entertainment/independent users (EIUs)) were collected. Videos were scored using the mDISCERN and Global Quality Score (GQS).

Results

Sixty-four videos were included. DISCERN scores significantly differed between POs (n=20, mean=4.35), HIWs (n=29, mean=2.97), and EIUs (n=15, mean=1.83). GQS also significantly differed between POs (n=20, mean=4.47), HIWs (n=29, mean=3.62), and EIUs (n=15, mean=2.5). Video characteristics significantly differed between groups, with most user engagement seen in EIUs.

Conclusion

POs and HIWs disseminate higher quality health information about diverticular disease on YouTube. The higher viewer engagement with EIUs is concerning, as these sources were found to have lower quality content. Although YouTube has the capability to provide valuable information on diverticulosis and diverticulitis, enhanced content screening is needed to ensure accuracy and validation.

## Introduction

More than 50% of Americans aged 60 years or older have diverticulosis, which is defined as an abnormal out-pouching of colonic mucosa [1]. When diverticula become inflamed, diverticulosis can progress to diverticulitis. This phenomenon occurs in approximately 20% of patients, with the incidence rising over the past 10 years, especially in patients aged 18-49 [1-3]. Upon receiving these diagnoses, patients, regardless of age, often turn to accessible online resources for additional information and to answer any questions they may have about their condition.

In fact, more than 70% of adults currently use online information to learn about their personal diagnoses and treatment options [4]. Approximately 20% of individuals aged 75 or older use the Internet to communicate with their physician, and about 80% use the Internet to seek health information, a marked increase from 57% in 2003 [5]. The continued expansion of social media further facilitates the acquisition, creation, and sharing of health-related patient resources virtually. There have been many studies evaluating the quality of disseminated health information across various social media websites [6-8]. YouTube is one of the world’s most popular media platforms, amassing over 1 billion users worldwide since its creation in 2005.

However, given the website’s variable guidelines for video publishers and inconsistent fact-checking protocols, the quality of information offered to patients has not been comprehensively explored [7]. A Google Transparency report in the final quarter of 2022 stated that just 1.8% of videos were removed for violating community guidelines on misinformation, with over two-thirds of these videos having 10 views or fewer [9]. As the prevalence of diverticulosis and diverticulitis in the United States is significant, it is imperative to understand the surplus of information available to patients. The aim of this study is to comprehensively assess the quality and engagement of posted video content pertaining to diverticulosis and diverticulitis on YouTube.

## Materials and methods

Search strategy

Four separate search queries were performed on YouTube using the targeted terms: (Diverticulosis), (Diverticulitis), (Acute Diverticulitis), and (Chronic Diverticulitis) on December 16, 2022. After applying the embedded filter to sort by “relevance,” the first 50 videos per search term were selected for cross-sectional analysis. Notably, this included videos labeled “From Health Sources.” This methodology was rooted in existing data that asserts 90% of YouTube users watch videos limited to the first few pages of results [10].

Two hundred videos were identified and then screened based on exclusion criteria, which included: (1) duplicate videos, (2) videos not in the English language, (3) videos not pertaining to either diverticulosis or diverticulitis, and (4) videos intended for healthcare professionals, specifically those discussing technical aspects of procedures, physical exam techniques, and other high-level surgical training videos.

Video classification

Videos were evaluated independently by two authors (Nair GA, Thang CJ). Video characteristics, including total view count, video duration, days since upload, video power index, view ratio, total likes, and public comments, were collected. The Video Power Index (VPI) was calculated by multiplying the view ratio by the like ratio and dividing by 100 (View ratio = views/day since upload, Like ratio = (likes * 100) / (likes + dislikes)). In accordance with previous literature, VPI was used as our primary proxy for assessing a video’s user engagement [11-14]. Videos were then classified into three groups based on the publishing entity: (1) Professional Organizations (POs), such as hospitals or universities, (2) Health Information Websites (HIWs), such as WebMD or Osmosis, and (3) Entertainment/Independent Users (EIUs), such as personal testimonies and blogs. These classifications were mutually agreed upon by the research team prior to data collection.

Reliability and quality assessment

Videos were assessed for reliability using the modified DISCERN (mDISCERN) rating tool adapted by Singh et al. [15,16] (Table 1). This tool assigns one point for each yes/no question with a minimum score of 0 and a maximum score of 5, corresponding with increasing reliability. The quality of each video was assessed using the Global Quality Scale (GQS) (Table 2). This scale ranges from 1 to 5, with a score of 1 point denoting poor quality, flow, and information relevance. Both tools are well-validated in previous literature [17].

**Table 1:** mDISCERN criteria for assessing video reliability. Reliability for each video was scored from 1 to 5 with 1 point given for each "Yes" answer. mDISCERN: modified DISCERN.

Questions Investigated
Is the video clear, concise, and understandable?
Are valid sources cited?
Is the information provided balanced and unbiased?
Are there additional sources of information for patient reference?
Does the video address topic uncertainty/controversy?

**Table 2:** Global Quality Scale (GQS) for assessing video quality. A GQS score from 1 to 5 was given to each video based on the associated GQS criteria.

Score Given	Score Interpretation
1	Poor quality and flow, most information missing, not useful for patients.
2	Generally poor quality and flow, some information mentioned but many important topics missing, limited usefulness to patients.
3	Moderate quality, suboptimal flow, some important information discussed adequately but other information discussed poorly, somewhat useful for patients.
4	Good quality, generally good flow, most important information mentioned, some topics not covered, overall useful for patients.
5	Excellent quality and flow, the highest level of usefulness for patients.

Statistical analysis

Statistical analysis was performed in RStudio. Interrater reliability for mDISCERN and GQS scores was calculated using the Intra-Class Correlation (ICC) to assess the reliability or consistency of agreement between raters or observers when evaluating the same set of items or subjects. An ICC of 0-0.2 indicated no agreement, 0.21 to 0.39 minimal agreement, 0.40 to 0.59 weak agreement, 0.60 to 0.79 moderate agreement, and ≥ 0.80 strong agreement, ensuring credibility and consistency between raters [18]. The F-test, also known as the ANOVA for ICC, was measured to determine whether the observed level of agreement between raters is statistically different from what would be expected by chance, with significance set to p < 0.05. All variables were tested for normality using the Shapiro-Wilk test. Differences between continuous variables across the three video categories (POs, HIWs, and EIUs) were assessed using analysis of variance for normally distributed variables and the Kruskal-Wallis test for skewed variables. Correlation analysis was again performed, using the Shapiro-Wilk normality test to assess normal distribution, with Spearman’s rank correlation rho used for non-parametric data. Spearman’s rank correlation rho can be positive or negative, indicating whether the variables are positively or negatively associated with each other. The value of rho indicates the strength of the correlation, ranging anywhere between 0 and 1. Rho values between 0 and 0.19 indicate a very weak correlation, 0.20 and 0.39 indicates a weak correlation, 0.40 and 0.69 indicates a moderate correlation, 0.70 and 0.89 indicates a strong correlation, and 0.90 and 1.00 indicates a very strong correlation. The single-score ICC was used to assess rater agreement for mDISCERN and GQS. The ICC values were evaluated as follows: values above 0.9 indicated excellent reliability, values between 0.75 and 0.9 indicated good reliability, and values between 0.5 and 0.75 indicated moderate reliability [19]. The confidence level was set at 95%, with significance considered as p < 0.05.

## Results

**Figure 1:**
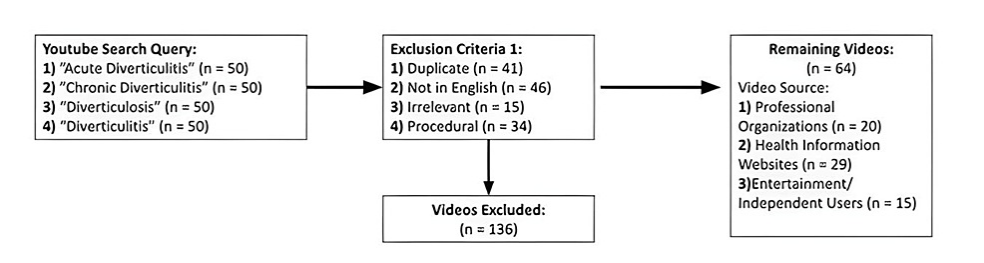
Search query, exclusion criteria, and included videos by source.

A total of 200 videos were initially identified, of which 136 were excluded, leaving 64 videos for study analysis (Figure 1). Twenty videos were classified as POs, 29 videos as HIWs, and 15 videos as EIUs. Collectively, the 64 selected videos had a total view count of 7,405,483, with a median view count of 47,922. The median duration of the selected videos was 6 minutes 23 seconds. Thirty-seven (57.8%) videos originated in the United States, six (9.4%) in the United Kingdom, five (7.8%) in Canada. Malaysia, Italy, Austria, Switzerland, and Germany each contributed one (1.6%) video. Eleven (17.2%) videos were from unspecified nations.

Inter-rater reliability for the mDISCERN and GQS assessment tools indicated strong agreement between the two raters. For the mDISCERN tool, the single score ICC was calculated to be 0.761, suggesting a moderate level of agreement between the raters' evaluations. The F-test (conducted to test the hypothesis that the true correlation is zero) showed a statistically significant result (F(63, 64) = 7.4, p < 0.001), indicating that the correlation is indeed greater than zero. Similarly, for the GQS tool, the single score ICC was found to be 0.788, again indicating a moderate level of agreement between the raters. The F-test also yielded a significant result (F(63, 63.5) = 8.57, p < 0.001), further confirming that the correlation between the raters is greater than zero. These findings ensure the credibility and consistency of the evaluations performed using these tools.

mDISCERN scores significantly differed (p < 0.001) between POs (4.35 ± 0.564), HIWs (2.97 ± 1.21), and EIUs (1.83 ± 0.116), with higher values observed in POs and HIWs. Similarly, GQS significantly differed (p < 0.001) between POs (4.47 ± 0.550), HIWs (3.62 ± 1.34), and EIUs (2.50 ± 1.15), with higher values seen in POs and HIWs. Video engagement, calculated using VPI, significantly differed between groups (p < 0.05). The highest engagement was observed in EIUs (114.66 ± 244.26), followed by HIWs (82.82 ± 141.28) and POs (16.66 ± 20.45). Higher mDISCERN and GQS scores were not directly correlated with any video engagement parameters except for duration (mDISCERN (p < 0.05, ρ = 0.28) and GQS scores (p < 0.05, ρ = 0.29)) (Tables 3-4).

**Table 3:** Comparison of video characteristics by publishing entity. P-value <0.05 indicates statistical significance. Numbers reported as mean (± SD).

Variables	Professional Organizations (POs) (n = 20)	Health Information Websites (HIWs) (n = 29)	Entertainment Sources/Independent Users (EIUs) (n = 15)	P-value (Kruskal-Wallis test)
DISCERN	4.35 (± 0.564)	2.97 (± 1.21)	1.83 (± 0.116)	p<0.001
GQS	4.47 (± 0.550)	3.62 (± 1.34)	2.5 (± 1.15)	p<0.001
Video Power Index	16.66 (± 20.45)	82.82 (± 141.28)	114.66 (± 244.26)	p=0.012
View Count	34390 (±41196)	106025 (±143686)	154147 (±200489)	p=0.010
View Ratio	17.83(± 21.45)	83.54(± 143.39)	117.68 (± 247.14)	p=0.011
Likes	270 (± 290)	1990 (± 3379)	3479 (± 5350)	p=0.004
Comments	34.2 (± 43.3)	130 (± 208)	438 (± 500)	p=0.003
Duration (seconds)	941 (± 1182)	469 (± 359)	493 (± 313)	p=0.894
Days Since Upload	2170.65 (± 1265.62)	1649.66 (± 1027.51)	2267.67 (± 1162.00)	p=0.111

**Table 4:** Correlation analysis between video characteristics and DISCERN and GQS scores. P-value < 0.05 indicates statistical significance. Numbers are reported as rho (p-value). GQS: Global Quality Scale.

Variables	DISCERN	GQS
View Count	-0.037 (0.772)	0.094 (0.462)
Video Power Index	-0.038 (0.764)	0.105 (0.409)
View Ratio	-0.036 (0.779)	0.106 (0.405)
Likes	-0.060 (0.636)	0.127 (0.317)
Comments	-0.112 (0.394)	0.037 (0.778)
Duration	0.280 (0.025)	0.288 (0.021)
Days Since Upload	-0.062 (0.626)	-0.119 (0.349)

## Discussion

Our analysis demonstrated that YouTube videos released by POs and HIWs disseminate higher quality and more reliable knowledge than videos from EIUs, as evidenced by higher mDISCERN and GQS scores. Videos from POs were generally longer, cited appropriate sources, and provided relevant details about diagnoses. Unfortunately, videos from POs and HIWs also had comparatively lower engagement parameters. The VPI was lower in both POs and HIWs when compared to EIUs, with marked differences seen between the VPI of POs and EIUs. This suggests that YouTube users watched and engaged more often with videos published by EIUs than those published by POs and HIWs. It is worth noting that many videos produced by POs and HIWs present more complex health information and generally target viewers with healthcare experience. However, video results are displayed similarly for all users, regardless of background or educational status, implying that there may be a subjective component discouraging laypeople from watching PO/HIW-produced videos. Overall, these results raise concerns that videos from more reputable publishers receive significantly less attention and engagement from viewers, particularly those produced by POs.

With the advent of smartphones, laptops, and other technological advances, online health information has never been more publicly accessible. Consequently, this poses challenges for patients in discerning reputable medical information. Disseminated medical advice on social media does not undergo a true peer-review process, and users who publish health misinformation often face no penalty for doing so [8]. Furthermore, users have the freedom to self-categorize their videos as educational or as medical advice without proper verification.

YouTube’s search ranking system is openly described on their website under the “How YouTube Works” tab. The process utilizes three main criteria to generate search results: relevance, engagement, and quality [20]. Relevance is determined largely by the video uploader’s tags, engagement by aggregate engagement signals such as total watch time for a video, and quality by an undefined mechanism that identifies which channels are more authoritative sources of information [20]. This system implies that videos with higher measures of engagement receive higher priority within the YouTube search algorithm. Therefore, viewers who watch videos produced by EIUs seemingly perpetuate a vicious cycle of published misinformation.

Another explanation for the observed results could be that the content from POs and HIWs is intended to cater more to academia, such as lectures, infographics, and recorded conferences. While both informative and reliable, these videos may be less attractive to the average viewer searching for personal inquiry rather than for professional or educational reasons [21]. This aligns with previous literature suggesting that public interest in YouTube videos is independent of publisher accreditation [22].

Based on this study’s findings, the importance of health literacy among the patient population cannot be understated. Specifically, low health literacy presents a challenge for patients seeking to educate themselves about their medical conditions by rendering them unable to accurately assess online information. This allows for low-quality information from EIUs to have a disproportionately negative impact on those with decreased health literacy. To address this, healthcare providers must directly aid patients in finding quality health information. Providers can implement this study's findings by distributing links to reputable videos and channels while simultaneously alerting patients to the misinformation present in content produced by EIUs. These resources need to balance reliability with average user comprehension, a responsibility that falls into the hands of public health organizations, healthcare systems, and educational organizations. Ultimately, without careful vetting of sources, YouTube is not currently reliable for obtaining quality health information about diverticulosis and diverticulitis. Our results are consistent with other studies investigating the quality of information on various health topics on YouTube [17, 23-24].

Some limitations are present in this study. First, this was a cross-sectional study that captured YouTube search results at a single time point. As the largest video-sharing website in the world, YouTube’s database is dynamic, with search results and posted content changing rapidly. Second, mDISCERN and GQS scores for each video relied on subjective grades from two reviewers. While we attempted to account for this by demonstrating high inter-rater reliability, we acknowledge the potential for variability. Lastly, given our academic background, the resulting conclusions may not be directly generalizable to all patient populations; however, future qualitative studies incorporating patient perspectives may help create a balance between reliable data and patient preferences.

## Conclusions

YouTube and other social media platforms will continue to be highly utilized resources for patients seeking to learn about commonly diagnosed conditions such as diverticulosis and diverticulitis. In general, users utilize video engagement parameters such as view count and number of likes to determine which videos they choose to watch. However, parameters like these are largely not reflective of information quality and reliability. Our findings emphasize the role of health care providers in guiding patients in finding accurate health information. They also highlight the responsibility of public health organizations to proactively create reliable patient-centered resources.
